# Climate forcing of an emerging pathogenic fungus across a montane multi-host community

**DOI:** 10.1098/rstb.2015.0454

**Published:** 2016-12-05

**Authors:** Frances C. Clare, Julia B. Halder, Olivia Daniel, Jon Bielby, Mikhail A. Semenov, Thibaut Jombart, Adeline Loyau, Dirk S. Schmeller, Andrew A. Cunningham, Marcus Rowcliffe, Trenton W. J. Garner, Jaime Bosch, Matthew C. Fisher

**Affiliations:** 1 Institute of Zoology, Zoological Society of London, Regent's Park, London NW1 4RY, UK; 2 Department of Infectious Disease Epidemiology, Imperial College London W2 1PG, UK; 3 Department of Life Sciences, Imperial College London, Silwood Park Campus, SL5 9PU, UK; 4 Computational and Systems Biology, Rothamsted Research, West Common, Harpenden, Hertfordshire AL5 2JQ, UK; 5 Université de Toulouse; UPS, INPT; EcoLab (Laboratoire Ecologie Fonctionnelle et Environnement), 118 route de Narbonne, 31062 Toulouse, France; 6 Department of Conservation Biology, Helmholtz Centre for Environmental Research - UFZ, Permoserstrasse 15, 04318 Leipzig, Germany; 7 Department of System Ecotoxicology, Helmholtz Centre for Environmental Research - UFZ, Permoserstrasse 15, 04318 Leipzig, Germany; 8 Museo Nacional de Ciencias Naturales, CSIC, Jose Gutierrez Abascal, 2 28006, Madrid, Spain

**Keywords:** climate change, chytridiomycosis, multi-host communities, epidemiology, mountain ecosystems, host × pathogen × environment interaction

## Abstract

Changes in the timings of seasonality as a result of anthropogenic climate change are predicted to occur over the coming decades. While this is expected to have widespread impacts on the dynamics of infectious disease through environmental forcing, empirical data are lacking. Here, we investigated whether seasonality, specifically the timing of spring ice-thaw, affected susceptibility to infection by the emerging pathogenic fungus *Batrachochytrium dendrobatidis* (*Bd*) across a montane community of amphibians that are suffering declines and extirpations as a consequence of this infection. We found a robust temporal association between the timing of the spring thaw and *Bd* infection in two host species, where we show that an early onset of spring forced high prevalences of infection. A third highly susceptible species (the midwife toad, *Alytes obstetricans*) maintained a high prevalence of infection independent of time of spring thaw. Our data show that perennially overwintering midwife toad larvae may act as a year-round reservoir of infection with variation in time of spring thaw determining the extent to which infection spills over into sympatric species. We used future temperature projections based on global climate models to demonstrate that the timing of spring thaw in this region will advance markedly by the 2050s, indicating that climate change will further force the severity of infection. Our findings on the effect of annual variability on multi-host infection dynamics show that the community-level impact of fungal infectious disease on biodiversity will need to be re-evaluated in the face of climate change.

This article is part of the themed issue ‘Tackling emerging fungal threats to animal health, food security and ecosystem resilience’.

## 1. Introduction

Climate change is likely to influence infectious disease dynamics, with many pathogens, especially those with complex life cycles or those infecting ectothermic hosts, predicted to change in severity or range as the earth continues to warm [1–3]. *Batrachochytrium dendrobatidis* (*Bd*), one of two fungi known to cause amphibian chytridiomycosis, is associated with the decline and extinction of amphibians worldwide [4,5]. In common with other infections, it is widely assumed that climate change has facilitated epizootics of chytridiomycosis, thus allowing *Bd* to establish in naïve ecosystems [6–8].

A link between climatic variables, host phenology (the timing of recurring natural phenomena) and the population-level impact of *Bd* has been investigated by both *in situ* [6,7] and *ex situ* [8–10] studies. However, the hypothesis that climate change dictates *Bd* infection dynamics in nature has not been proven. This is because the key *in situ* studies to date have confounded two variables, pathogen introduction and environmental variation [6,7], and the required longitudinal epidemiological studies in established diseased ecosystems have not yet been undertaken [11,12]. Further, *Bd* is a generalist pathogen [13], exhibiting broad variation in its ability to infect and cause disease across species [14]. Within multi-species amphibian assemblages, different host species exhibit a range of responses to pathogen exposure. These responses include resistance to infection (diluters of infection), infection tolerance (reservoirs of infection) and variation in susceptibility to lethal disease [15,16]. Yet studies to date have focused on the most readily infected species assuming homogeneous host response over time, and data that addresses the medium to long-term temporal impact of *Bd* across all members of a host community in concert with local climatic data is absent.

Climate variation is pronounced in montane systems, and it is in these environments that epizootics of chytridiomycosis predominantly occur [17–20] presenting an opportunity to untangle the complex relationship between disease dynamics and climate. One such environment, the Pyrenean mountain range, contains many lakes housing multi-species amphibian assemblages within an expanding epizootic of *Bd* infection [20]*.* Across the core outbreak region of the Western Pyrenees, the midwife toad (*Alytes obstetricans*) experiences annual mass mortality due to chytridiomycosis. Two other anuran species also commonly breed in this area, the common toad (*Bufo spinosus,* previously known as *Bufo bufo* [21]) and the common frog (*Rana temporaria*). Across Europe, the prevalence of *Bd* within affected common toads is usually low [14], with the majority of individuals tolerating infection [22]. In comparison, the common frog is considered to be resistant to *Bd* infection [14,23], and until now there has been no evidence of disease in this species. As a result of the differences that these three species exhibit in their response to exposure to *Bd*, they constitute an ideal study-system for exploring the temporal trends in pathogen infection at a community level. To understand the inter-relationship between infection, community and climate, we investigated how seasonality, specifically the timing of spring thaw, in this montane ecosystem affects infection dynamics both at the present and when extrapolated into the future using downscaled outputs of global climate change models.

## 2. Results and discussion

**Figure 1.**
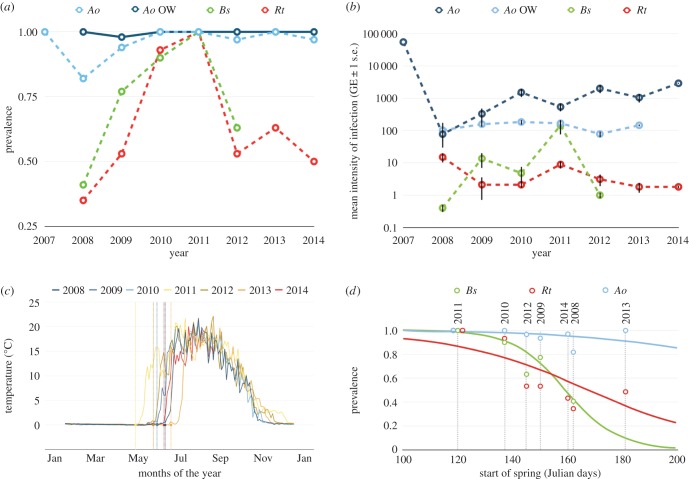
(*a*) Temporal change in prevalence of infection for OW tadpole and metamorphic *Alytes obstetricans* (*Ao*) and metamorphic *Bufo spinosus* (*Bs*) and *Rana temporaria* (*Rt*)*;* (*b*) temporal change in intensity of infection; (*c*) seasonal changes in water temperature and timing of spring onset in Lac Arlet; (*d*) relationship between spring onset and the prevalence of *Bd* infection across all species.

We monitored amphibians at a key remote infected site, Lac Arlet (altitude 1986 m.a.s.l.), in the French Pyrenees, over 7 years. Across this period, we found a robust temporal link between disease dynamics (figure 1*a*,*b*) and the timing of spring (the date of lake ice thaw) across this community of amphibians (figure 1*c*,*d*). We uncovered a significant effect of the onset of spring on the prevalence of *Bd* infection in both *B. spinosus* (slope = −0.1000; *z* = −4.715, d.f. = 149, *p* < 0.001; adjusted *R*^2^ = 0.19) and *R. temporaria* (slope = −0.039, *z* = −4.356, d.f. = 207, *p* < 0.001; adjusted *R*^2^ = 0.08), with early spring onset resulting in a higher prevalence of *Bd* infection (figure 1*d*). Such an effect was observed in the highly susceptible *A. obstetricans* metamorphs, but the difference in prevalence was not significant (*p* = 0.09), and a high prevalence of infection was maintained over the years. The timing of the end of season showed little variation between years (figure 1*c* and table 1), with the corollary that the length of the amphibian activity period was increased in years with an early spring onset.

**Table 1.** The timing, in Julian days, of the start of spring and the end of season.

year	start of spring	end of season	days of activity
2008	162	302	140
2009	150	n.a.	n.a.
2010	137	298	161
2011	120	298	178
2012	145	302	157
2013	181	308	127
2014	160	309	149

We found the intensity of infection was significantly greater in the metamorphs of *A. obstetricans* than either *B. spinosus* or *R. temporaria* in all years (electronic supplementary material, table S1*a*), and year was a significant predictor of infection intensity in all three species (electronic supplementary material). It is possible that an increase in infection load in the highly parasitized *Alytes* could contribute to the changes in prevalence seen in the other two species, but we found no significant association between *Alytes* infection load and the prevalence of infection in either *B. spinosus* or *R. temporaria* (*p* = *0.68*; *0.88,* respectively).

**Figure 2.**
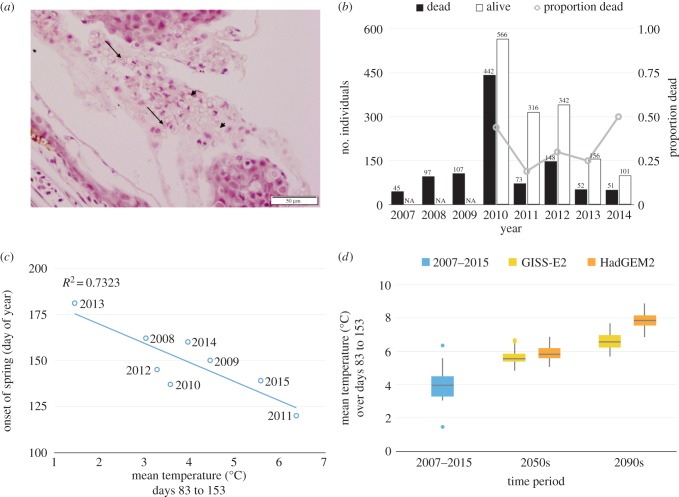
(*a*) Histology slide showing a section from the hind legs of a deceased *R. temporaria* metamorph demonstrating clear evidence of the disease chytridiomycosis. The two long arrows point to two of many sporangia full of zoospores, and the two short arrows point to empty sporangia cases (once zoospores have burst out), both embedded with the upper skin layers; (*b*) counts of live and dead *A. obstetricans* metamorphs over time with the number of dead (black) and alive (white) *A. obstetricans* metamorphs encountered. No ‘alive’ counts were made for years 2007–2009; (*c*) Relationship between air temperature and the onset of spring at Lac Arlet and (*d*) observed and future predictions of air temperatures over Julian days 83–153 at Lac Arlet.

The fact that a species previously resistant to infection and disease alters in different environmental/climatic contexts illustrates the importance of understanding community-level dynamics when considering the impacts of infections. Our data show that a species thought to be highly resistant to infection and disease can suffer mortality (figure 2*a*); prior to the current study, *R. temporaria* had rarely been found to be infected with *Bd* [14] and had never been observed to suffer chytridiomycosis despite widespread surveillance across Europe. However, we detected widespread infection in this species, along with mortality due to chytridiomycosis (figure 2*a*), when spring onset was early. We also found concurrent temporal changes in the prevalence of *Bd* infection in the more susceptible species, *B. spinosus,* which became locally extinct in 2013 (figure 1*a*,*b*). We believe that the abrupt decline of *B. spinosus* was disease-driven, owing to the high prevalence of infection and widespread mortality detected in metamorphs in the years leading to its disappearance. The number of *A. obstetricans* over-wintered (OW) larvae has also declined steadily at Lac Arlet during the seven-year course of our study, alongside high recorded mortality (figure 2*b* and table 2), showing that mortality due to chytridiomycosis is leading to synchronous multi-species declines across this site.

**Table 2.** Visual estimates of live amphibian abundance+less than 100; ++100–1000; +++ more than 1000.

year	*Ao* OW tadpoles	*Ao* Mets	*Rt* Mets	*Bs* Mets
2008	+++	++	+++	+++
2009	+++	++	+++	+++
2010	+++	++	+++	++
2011	+++	++	+++	++
2012	++	++	+++	+
2013	+	++	+++	0
2014	+	++	+++	0

Our data suggest that the high degree of variation in species susceptibility to infection may lead them to play different roles in the transmission of infection. Further, the extent to which infection spreads inter-specifically within the system may be greatly influenced by the timing of ice thaw, and hence the period of winter inactivity that these species experience. At the altitude of Lac Arlet (approx. 2000 m), *A. obstetricans* tadpoles over-winter for a number of years before completing development. The tadpole stage of the majority of amphibians rarely suffer mortality or visible effects of *Bd* infection [24,25] and *A. obstetricans* tadpoles harbour *Bd* infection at high prevalence and infection intensity (figure 1*a*,*b*). High levels of infection, a long period of infectiousness and low mortality rates are all key parameters in determining the rate of spread of infectious disease and suggest that overwintering *A. obstetricans* larvae could therefore play a major role in the maintenance of *Bd* across seasons, as has been proposed previously for other systems [19,26]. The yearly changes in infection prevalence that we detected in hosts that are less susceptible to infection could imply that the density of one generally heavily infected species, *A. obstetricans,* drives infection levels within this system. We detected the highest abundance of *A. obstetricans* metamorphs in 2010 and in that year we also saw the highest proportional mortality, with almost half of all *A. obstetricans* individuals encountered being dead. It is possible that the sharp increase in prevalence of infection in 2010 that we detected in both *B. spinosus* and *R. temporaria* was a direct result of the observed increase in the number of infected *A. obstetricans* in that year. However, there was no significant association between the number of deceased *A. obstetricians* mortalities seen each year and the prevalence of infection in either species. Further, the prevalence of infection in both *B. spinosus* and *R. temporaria* continued to increase the following year (figure 1*a*), despite a decrease in the abundance of *A. obstetricans* (figure 2*b*)*.* This contraindicates the hypothesis that the density of *A. obstetricans* is the sole driver of force of infection upon sympatric species.

To assess the impact of climate change on onset of spring, the optimal time period over which mean air temperature is most closely related to time of spring thaw was estimated (electronic supplementary material, figure S1). For 2007–2015, mean temperature over days 83–153 (a 70 day period centred on day 118 of the year) was found to be the best predictor of onset of spring, explaining 73% of the variance. For every 1° increase in the mean temperature over this time, onset of spring is estimated to be 10 days earlier (figure 2*c*).

We then used the LARS-WG weather generator [27] as a downscaling technique to generate local-scale climate scenarios for the site, based on projections from global climate models (GCMs) from the CMIP5 multi-model ensemble under the representative concentration pathway (RCP) 8.5 (greenhouse gas emissions continue rising over twenty-first Century) [28] and modelled using local weather data (figure 2*c*). To capture uncertainty in the CMIP5 climate projections, we selected two GCMs, GISS-E2 and HadGEM2, with low and high climate sensitivities, respectively, which thus predict lesser and greater amounts of warming for the region. The predictions all indicate that an early onset of spring in the western Pyrenees will become commonplace by the 2050s due to global warming (figure 2*d*). Over days 83–153 of the year, the mean daily temperature is projected to be around 5.5°C (5.4–5.8°C) by GISS-E2 and 5.8°C (5.6–6.2°C) by HadGEM2 in the 2050s (medians and inter-quartile ranges of 100 years plausible weather generated by LARS-WG; figure 2*d*). These correspond, under the current relationship, with estimated median onsets of spring at day 133 or 130. By the 2090s, the median GISS-E2 scenario for this time is 6.5°C, potentially indicating an onset of spring around day 122, close to that of 2011, the earliest melt and highest prevalence year observed in this study. The median HadGEM2 2090s scenario is 7.8°C; because this is out of the range of observed temperatures (with the rest of the year also being substantially warmer), we make no estimated projection of onset of spring under this scenario. LARS-WG output across the winter months for the 2090s for HadGEM2 suggests that daily mean temperature will rarely drop below 0°C (daily minimum temperature may be below freezing for three months, compared to currently around six months), which may lead to a short, punctuated or non-existent duration of ice cover (electronic supplementary material, figure S2). If the trends that we have established here continue, increases in amphibian activity periods are predicted to correspond to increases in infection levels across these species which could have profound effects on populations of hosts we currently consider to be resistant or tolerant to infection. An onset of spring at day 133, under the currently estimated relationship, suggests a prevalence of *Bd* infection in *B. spinosus* above 0.9. If the length of season continues to drive the prevalence in the same manner with all other influences remaining the same, then prevalence of this magnitude could be frequent from the 2050s. Further years of data would of course allow stronger assessment of this relationship. The range of possibilities for the climate in the 2090s includes the potential for a drastic reduction in lake ice cover, which could greatly change the epidemiology of this infection within this system.

The mechanisms underpinning the seasonal forcing of infection that we observe are unknown. A broad range of biotic and abiotic factors are influenced by seasonality, and it is probable that many factors acting on both the host and the pathogen are driving the seasonal prevalence of infection in the amphibians studied. On the one hand, abiotic drivers include increased temperature volatility causing a reduction in temperature acclimation of host resistance to infection [29], and patterns of disease are known to be also exacerbated as hosts are moved outside of their temperature norms [9]. On the other hand, biotic factors such as the density and species complement of aquatic microfauna present at our research site are known to modulate the risk of infection [30]. Aquatic microfauna have seasonal peaks and dips in abundance throughout the spring and summer corresponding to the local availability of nutrients [31], therefore, changes in the timing of the seasons may affect the abundance of the microfauna, impacting the removal of *Bd* zoospores and force of infection. When complex biotic and abiotic drivers such as these interact, nonlinear responses are expected and the uncertainty associated with predicting future trends in this, and other montane systems, will likely be high. However, regardless of the underlying mechanisms, our findings robustly show that annual changes in the timing of spring thaw drives synchronous infection dynamics in *B. spinosus* and *R. temporaria*, despite previous research indicating a clear difference in susceptibility to *Bd* infection between these species [14,32]. More broadly, we have shown that failing to include environmental information may undermine our understanding of how pathogens spread and persist within host communities. Identifying differences in infection levels across species under different environmental conditions is therefore central to understanding disease ecology within multi-host communities, especially where changes in climate are predicted to exacerbate the impact of emerging infectious disease leading to further losses of biodiversity.

## 3. Material and methods

**Figure 3.**
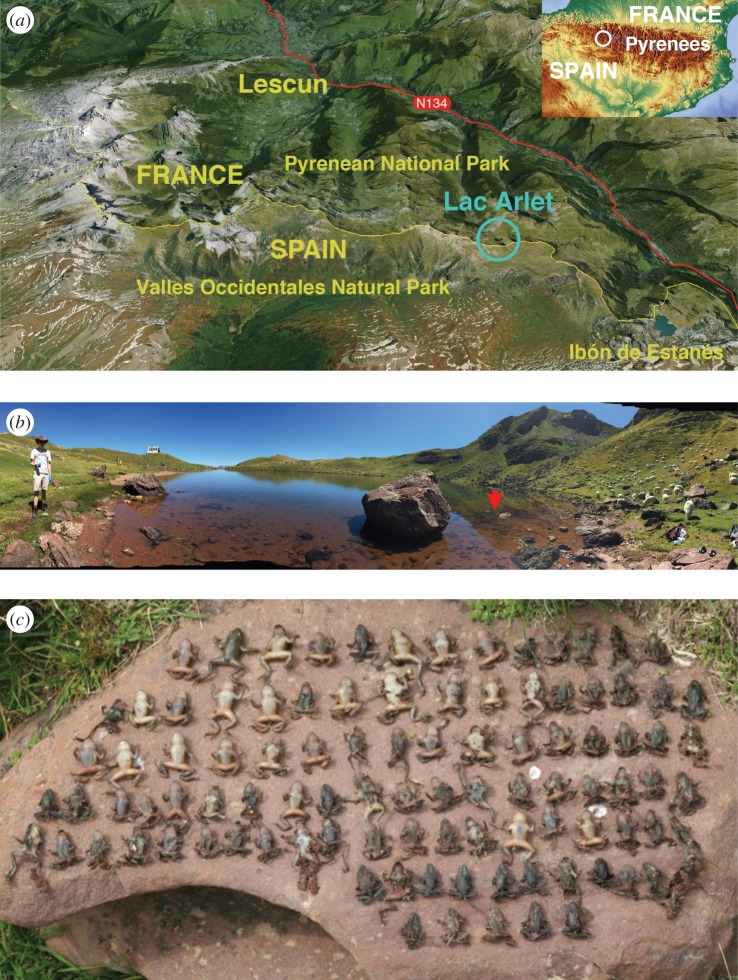
(*a*) Location of the study site (*b*) Lac Arlet showing the position of temperature datalogger (red arrow). (*c*) Mass mortalities of midwive toads *Alytes obstetricans* caused by *Batrachochytrium dendrobatidis* lineage *Bd*GPL at Lac Arlet.

The study was conducted at Lac Arlet (Longitude: 0°36'54.12″ W, Latitude: 42°50'24.20″ N) in the Pyrenean National Park between 2007 and 2014 (with dates of thaw up to 2015). This lake sits at an altitude of 1986 m and encompasses an area of 2.7 ha (figure 3).

### (a) Prevalence and intensity of *Bd* infection

All three species of amphibian present at Lac Arlet were sampled: emerging metamorphs of *B. spinosus* and *R. temporaria* and two life stages of *A. obstetricans*, OW tadpoles and recent metamorphs; OW tadpoles are those which have spent at least one winter in the lake before completing metamorphosis. To sample the animals, sterile rayon-tipped swabs (MWE medical wire) were gently rotated (10 times per individual) over the mouthparts of OW tadpoles, and used to swab the hind legs, feet and pelvic patch (five swipes per area, with the swab rotated between each area) of each recent metamorph. All swabs were stored in dry tubes at 4°C until processing. Sampling of tadpoles was conducted each year in July. Metamorphs were sampled in August during the height of metamorphic emergence. In 2007, toe clips were taken from metamorphic *A. obstetricans* instead of skin swabs; a 2–3 mm clip was cut from a single hind toe using a sterile scalpel blade. These toe clips were fixed in 70% ethanol.

We followed the protocol of Boyle *et al*. [33], to quantify *Bd* prevalence and intensity of infection, as assessed by quantitative PCR (qPCR). To avoid inhibition, all extractions were diluted 1 : 10 prior to qPCR; therefore, results were multiplied by 10 in order to determine the true value. We defined infection intensity as the number of *Bd* zoospore genomic equivalents (GE) per swab. All samples were run in duplicate, and a sample was assigned a positive reading if both wells amplified and an average estimate of 0.1 GE or above was produced when comparing the sample to the curve generated by the standards. Samples were repeated up to three times if only one well amplified, after which time they were assigned a negative reading if both wells failed to amplify.

### (b) Population counts

Counts of newly emerged live and dead metamorphic *A. obstetricans* were conducted twice (approx. one week apart) during each metamorph emergence period each year from 2010 to 2014. Searches were carried out around the entire circumference of the lake, from the shallows of the lakes (less than 0.5 m in depth) to a 1.5 m distance from the water's edge onto dry land, including under movable rocks. Any deceased metamorphs found were removed so as not to be counted a second time on subsequent visits. We assumed that live animals would either disperse from the water's edge within a few days of emergence or die, and would therefore not be counted again in subsequent live estimates. Approximate counts (less than 100; 100–1000; more than 1000) were made for both newly emerged *R. temporaria* and *B. spinous* metamorphs each year, during the above surveying time. This measure was also applied to *A. obstetricans* OW larvae by scanning a 2 m area of the water, from the water's edge.

### (c) Diagnosis of chytridiomycosis in *Rana temporaria*

Owing to the high prevalence of *Bd* that we observed infecting *R. temporaria*, we decided to investigate whether individuals were suffering chytridiomycosis due to *Bd* infection, something which has not been shown before. Recently deceased, fresh *R. temporaria* metamorphs found around the edge of the lake were collected and fixed in 10% neutral buffered formalin. After fixation, the lower half of the each body (including pelvic region) and the front legs were processed for histopathological examination. Three levels per section were prepared, using a standard haematoxylin and eosin stain. Histological sections were examined microscopically to determine the presence or absence of the disease chytridiomycosis.

### (d) Determining length of season

Lac Arlet water temperature was measured every half an hour throughout the study period using a data logger (HOBO Water Temperature Pro v2 Data Logger – U22-001). The logger was positioned 2 m from the shore, approximately half a meter below the water surface, attached to a large rock with non-perishable wire. For each year, the date of the onset of spring and the date of the presumed end of the amphibian active season was recorded. The onset of spring was defined as the first day of the year (00:00–23:59) with a mean water temperature above 1°C, which remained so until the winter. The end of the active season was defined as the first day in the second half of the year when the mean water temperature dropped below 5°C, as European amphibians will often enter hibernation around this temperature [34]. This allowed the length of the active season (total number of possible ‘active days’ for amphibians) to be calculated by subtracting the Julian date of the onset of spring from the Julian date of the end of season.

### (e) Establishing the relationship between air temperature and lake thaw (onset of spring)

To project the impact of rising temperatures, we first quantified a linear relationship between air temperature and time of thaw, which corresponds to onset of spring for this ecological system. This is consistent with findings from other studies showing air temperature to be highly informative for thawing of many lakes [35], even if temperatures are measured some distance from the lake. To establish this relationship for this site, daily weather data (maximum and minimum temperatures, precipitation) were obtained from the nearest meteorological station to Lac Arlet, Canfranc Los Arañones, 13 km southeast of Lac Arlet. This is situated at 1160 m.a.s.l., 826 m below Lac Arlet. Hence, a correction of −5.29°C was applied to the air temperatures to account for tropospheric temperature decrease with altitude, in accordance with previous studies [36]. These adjusted temperature data along with daily precipitation for 1995–2015 were also used as baseline input for LARS-WG.

### (f) Generating local-scale climate scenarios

To find the optimal time period over which these air temperatures are most predictive for ice-thawing time, and to quantify that relationship, linear regressions were performed between air temperature and thaw dates for 2007–2015 inclusive. Mean daily air temperature was calculated over time periods of lengths varying from 21–81 days in 10-day increments, centred on days 30–170 of the year. The time period with the best predictive power for onset of spring, as judged by *R*^2^-value and standard model checking plots of the linear regression, was chosen. Analysis was performed in R v. 3.2.3.We used the LARS-WG weather generator as a downscaling technique [27] to generate local-scale climate scenarios, based on climate projections from GCMs from the CMIP5 multi-model ensemble used in the latest IPCC Assessment Report 5 (AR5) [28]. To capture uncertainty in the CMIP5 climate projections, we selected two GCMs with low, (GISS-E2), and high, (HadGEM2), climate sensitivities [37], which thus predict lesser and greater degrees of warming for this region. This allowed us to quantify uncertainty in predictions of the onset of spring under climate change. We generated 100 years of daily plausible weather for the periods 2050s and 2090s under RCP 8.5. The mean temperatures across the time period ascertained as most predictive for lake thaw were calculated from each of the 100 years of synthetic daily weather, for each GCM and time period combination.

### (g) Statistical analysis

All statistical analyses were carried out using the statistical software package R v. 3.2.3. All *Bd* DNA values (GE) were rounded to the nearest whole number and treated as count data. Any value of 0.1–0.9 was assigned a value of 1. Negative binomial regression models (function glm.nb from the R-package MASS) were used to look for any differences in the intensity of infection (GE values) between years and species. Likelihood ratio tests were used to assess the significance of predictor variables and of differences between factor levels within predictors. Where more than three factor levels remained significant, Tukey post-hoc tests (function glht from the package multcomp) were applied to allow pairwise comparisons. Fisher's exact test was used to compare differences in the prevalence of infection in each of *A. obstetricans* metamorphs and tadpoles, over the years 2007 (2008 for tadpoles) to 2014; in *B. spinosus* compared to *R. temporaria* over the years 2008–2012 and between each of *B. spinosus* and *R. temporaria* compared to *A. obstetricans* metamorphs over the years 2008–2014 (2008–2012 for *B. spinosus*). Pearson Correlation tests were used to determine whether there was a correlation between the prevalence of infection in both *B. spinosus* and *R. temporaria* and, (i) the mean infection intensity (GE) in *A. obstetricans* metamorphs, (ii) the number of dead *A. obstetricans* metamorphs encountered each year.

Using the temperature data, we were able to determine the date of the spring onset for all years apart from 2010, owing to a failure of the datalogger resulting in missing data. However, a strong association was seen between the onset of spring and the date at which the first *A. obstetricans* metamorph was seen in the years excluding 2010 (*t* = 7.724; *p* = 0.005, adjusted *R*^2^ = 0.94). We used this strong association to predict the onset of spring in 2010 and included the predicted value in all further analyses. The onset of season (mean Julian days = 150, s.d. = 20) varied more than the end of season (mean Julian days = 302, s.d. = 4); therefore, the onset of spring is the measure which primarily dictates season length. For this reason, we used spring onset as a proxy to assess changes in season length. Generalized Linear Models (GLM) using a binomial response (logistic regression) were used to determine whether there was a relationship between the prevalence of infection in all three species and the onset of spring each year. Likelihood ratio tests were used to assess the significance of these effects. A generalized adjusted *R*^2^ was calculated to assess the predictive power of each model [38].

## Supplementary Material

Supplementary information

## Data accessibility

Data available from the Dryad Digital Repository: http://dx.doi.org/10.5061/dryad.r7825.

## References

[1] Harvell CD, Mitchell CE, Ward JR, Altizer S, Dobson AP, Ostfeld RS, Samuel MD 2002 Climate warming and disease risks for terrestrial and marine biota. Science 296, 2158–2162. (10.1126/science.1063699) 12077394

[2] Epstein P 2010 The ecology of climate change and infectious diseases: comment. Ecology 91, 925–928. (10.1890/09-0761.1) 20426350

[3] Rodó X et al. 2013 Climate change and infectious diseases: can we meet the needs for better prediction? Clim. Change 118, 625–640. (10.1007/s10584-013-0744-1)

[4] Stuart SN, Chanson JS, Cox NA, Young BE, Rodrigues AS, Fischman DL, Waller RW 2004 Status and trends of amphibian declines and extinctions worldwide. Science 306, 1783–1786. (10.1126/science.1103538) 15486254

[5] Fisher MC, Garner TWJ, Walker SF 2009 Global emergence of *Batrachochytrium dendrobatidis* and Amphibian Chytridiomycosis in space, time, and host. Annu. Rev. Microbiol. 63, 291–310. (10.1146/annurev.micro.091208.073435) 19575560

[6] Pounds AJ et al. 2006 Widespread amphibian extinctions from epidemic disease driven by global warming. Nature 439, 161–167. (10.1038/nature04246) 16407945

[7] Bosch J, Carrascal LM, Duran L, Walker S, Fisher MC 2007 Climate change and outbreaks of amphibian chytridiomycosis in a montane area of Central Spain; is there a link? Proc. R. Soc. B 274, 253–260. (10.1098/rspb.2006.3713) PMC1685858 17148254

[8] Voyles J, Johnson LR, Briggs CJ, Cashins SD, Alford RA, Berger L, Skerratt LF, Speare R, Bree Rosenblum E 2012 Temperature alters reproductive life history patterns in *Batrachochytrium dendrobatidis*, a lethal pathogen associated with the global loss of amphibians. Ecol. Evol. 2, 2241–2249. (10.1002/ece3.334) 23139882 PMC3488674

[9] Ribas L et al. 2009 Expression profiling the temperature-dependent amphibian response to infection by *Batrachochytrium dendrobatidis*. PLoS ONE 4, e8408 (10.1371/journal.pone.0008408) 20027316 PMC2794374

[10] Garner TWJ, Rowcliffe JM, Fisher MC 2011 Climate change, chytridiomycosis or condition: an experimental test of amphibian survival. Glob. Change Biol. 17, 667–675. (10.1111/j.1365-2486.2010.02272.x)

[11] Rohr JR, Raffel TR 2010 Linking global climate and temperature variability to widespread amphibian declines putatively caused by disease. Proc. Natl Acad. Sci. USA 107, 8269–8274. (10.1073/pnas.0912883107) 20404180 PMC2889522

[12] Rohr JR, Raffel TR, Romansic JM, McCallum H, Hudson PJ 2008 Evaluating the links between climate, disease spread, and amphibian declines. Proc. Natl Acad. Sci. USA 105, 17 436–17 441. (10.1073/pnas.0806368105) PMC2582253 18987318

[13] Olson DH, Aanensen DM, Ronnenberg KL, Powell CI, Walker SF, Bielby J, Garner TWJ 2013 Mapping the global emergence of *Batrachochytrium dendrobatidis*, the amphibian chytrid fungus. PLoS ONE 8, e56802 (10.1371/journal.pone.0056802) 23463502 PMC3584086

[14] Baláž V et al. 2014 Assessing risk and guidance on monitoring of *Batrachochytrium dendrobatidis* in Europe through identification of taxonomic selectivity of infection. Conserv. Biol. 28, 213–223. (10.1111/cobi.12128) 24033675

[15] Bielby J, Bovero S, Angelini C, Favelli M, Gazzaniga E, Perkins M, Sotgiu G, Tessa G, Garner TWJ 2013 Geographic and taxonomic variation in *Batrachochytrium dendrobatidis* infection and transmission within a highly endemic amphibian community. Divers. Distrib. 19, 1153–1163. (10.1111/ddi.12085)

[16] Doddington BJ, Bosch J, Oliver JA, Grassly NC, Garcia G, Schmidt BR, Garner TWJ, Fisher MC 2013 Context-dependent amphibian host population response to an invading pathogen. Ecology 94, 1795–1804. (10.1890/12-1270.1) 24015523

[17] Bosch J, Martínez-Solano I, García-París M 2001 Evidence of a chytrid fungus infection involved in the decline of the common midwife toad (*Alytes obstetricans*) in protected areas of central Spain. Biol. Conserv. 97, 331–337. (10.1016/S0006-3207(00)00132-4)

[18] Whiles MR et al. 2006 The effects of amphibian population declines on the structure and function of Neotropical stream ecosystems. Front. Ecol. Environ. 4, 27–34. (10.1890/1540-9295(2006)004%5B0027:TEOAPD%5D2.0.CO;2)

[19] Briggs CJ, Knapp RA, Vredenburg VT 2010 Enzootic and epizootic dynamics of the chytrid fungal pathogen of amphibians. Proc. Natl Acad. Sci. USA 107, 9695–9700. (10.1073/pnas.0912886107) 20457916 PMC2906864

[20] Walker SF et al. 2010 Factors driving pathogenicity vs. prevalence of amphibian panzootic chytridiomycosis in Iberia. Ecol. Lett. 13, 372–382. (10.1111/j.1461-0248.2009.01434.x) 20132274

[21] Recuero E et al. 2012 Multilocus species tree analyses resolve the radiation of the widespread *Bufo bufo* species group (Anura, Bufonidae). Mol. Phylogenet. Evol. 62, 71–86. (10.1016/j.ympev.2011.09.008) 21964513

[22] Garner TWJ, Walker S, Bosch J, Leech S, Rowcliffe JM, Cunningham AA, Fisher MC 2009 Life history trade-offs influence mortality associated with the amphibian pathogen *Batrachochytrium dendrobatidis*. Oikos 118, 783–791. (10.1111/j.1600-0706.2008.17202.x)

[23] Bielby J, Fisher MC, Clare FC, Rosa GM, Garner TWJ 2015 Host species vary in infection probability, sub-lethal effects, and costs of immune response when exposed to an amphibian parasite. Sci. Rep.-UK 5, 10828 (10.1038/srep10828) PMC4448222 26022346

[24] Briggs CJ, Vredenburg VT, Knapp RA, Rachowicz LJ 2005 Investigating the population-level effects of chytridiomycosis: an emerging infectious disease of amphibians. Ecology 86, 3149–3159. (10.1890/04-1428)

[25] Bradley GA, Rosen PC, Sredl MJ, Jones TR, Longcore JE 2002 Chytridiomycosis in native Arizona frogs. J. Wildl. Dis. 38, 206–212. (10.7589/0090-3558-38.1.206) 11838218

[26] Rachowicz LJ, Vredenburg VT 2004 Transmission of *Batrachochytrium dendrobatidis* within and between amphibian life stages. Dis. Aquat. Organ. 61, 75–83. (10.3354/dao061075) 15584413

[27] Semenov MA, Stratonovitch P 2010 Use of multi-model ensembles from global climate models for assessment of climate change impacts. Clim. Res. 41, 1–14. (10.3354/cr00836)

[28] Stocker T 2014 Climate change 2013, the physical science basis: Working Group I contribution to the Fifth assessment report of the Intergovernmental Panel on Climate Change. Cambridge, UK: Cambridge University Press.

[29] Raffel TR, Romansic JM, Halstead NT, McMahon TA, Venesky MD, Rohr JR 2013 Disease and thermal acclimation in a more variable and unpredictable climate. Nat. Clim. Change 3, 146–151. (10.1038/nclimate1659)

[30] Schmeller DS et al. 2014 Microscopic aquatic predators strongly affect infection dynamics of a globally emerged pathogen. Curr. Biol. 24, 176–180. (10.1016/j.cub.2013.11.032) 24374305

[31] Scheffer M, Rinaldi S, Kuznetsov YA, van Nes EH 1997 Seasonal dynamics of *Daphnia* and algae explained as a periodically forced predator-prey system. Oikos 80, 519–532. (10.2307/3546625)

[32] Bosch J, Rincon PA 2008 Chytridiomycosis-mediated expansion of *Bufo bufo* in a montane area of Central Spain: an indirect effect of the disease. Divers. Distrib. 14, 637–643. (10.1111/j.1472-4642.2007.00461.x)

[33] Boyle DG, Boyle DB, Olsen V, Morgan JAT, Hyatt AD 2004 Rapid quantitative detection of chytridiomycosis (*Batrachochytrium dendrobatidis*) in amphibian samples using real-time Taqman PCR assay. Dis. Aquat. Organ. 60, 141–148. (10.3354/dao060141) 15460858

[34] Reading CJ 1998 The effect of winter temperatures on the timing of breeding activity in the common toad *Bufo bufo*. Oecologia 117, 469–475. (10.1007/s004420050682) 28307671

[35] Thompson R, Ventura M, Camarero L 2009 On the climate and weather of mountain and sub-arctic lakes in Europe and their susceptibility to future climate change. Freshwater Biol. 54, 2433–2451. (10.1111/j.1365-2427.2009.02236.x)

[36] Thompson R et al. 2005 Quantitative calibration of remote mountain-lake sediments as climatic recorders of air temperature and ice-cover duration. Arct. Antarct. Alp. Res. 37, 626–635. (10.1657/1523-0430(2005)037%5B0626:QCORMS%5D2.0.CO;2)

[37] Semenov MA, Stratonovitch P 2015 Adapting wheat ideotypes for climate change: accounting for uncertainties in CMIP5 climate projections. Clim. Res. 65, 123–139. (10.3354/cr01297)

[38] Heinzl H, Mittlbock M 2003 Pseudo R-squared measures for Poisson regression models with over- or underdispersion. Comput. Stat. Data Anal. 44, 253–271. (10.1016/S0167-9473(03)00062-8)

