# Supplementary material for: Climate forcing of an emerging pathogenic fungus across a montane multi-host community
Source: Philos Trans R Soc Lond B Biol Sci. 2016 Dec 5;371(1709):20150454. doi: 10.1098/rstb.2015.0454 (PMC5095533; doi:10.1098/rstb.2015.0454)
Supplement: Supplementary information [file rstb20150454supp1.docx]

**Supplementary Information**

**Extended results**

We detected a high prevalence of *Bd* infection (between 82% and 100%) in both the over wintered (OW) larval, and metamorphic life stages of *A. obstetricans* throughout the study period. In contrast, *Bd* prevalence in both *B. spinosus* and *R. temporaria* metamorphs varied over the years (41 to 100% and 35 to 100% respectively); a significantly lower prevalence of infection was detected in both species when compared to *A. obstetricans* in 2008 and 2012 (Fisher’s exact test, *p*=0.023), and in *R. temporaria* compared to *A. obstetricans* in 2009, 2013 and 2014 (Fisher’s exact test, 2009 *p*<0.001; 2013 *p* = 0.005; 2014 *p*<0.001) (Figure 1a & b).

Year was a significant predictor of infection intensity in all three species: *A. obstetricans* metamorphs (Chi-sq = 183, df =6, *p*<0.001) and OW-tadpoles (Chi-sq = 16, df =4, *p* = 0.003), *B. spinosus* metamorphs (Chi-sq = 109, df =4, *p*<0.001), and *R. temporaria* metamorphs (chi-sq = 49, df =5, *p* <0.001) (Figure 1d). The infection intensity detected in both *R. temporaria* and *B. spinosus* was significantly lower than that detected in *A. obstetricans* in all years (Supplementary Data Table 1A); and the infection intensity detected in *B. spinosus* was significantly lower than that detected in *R. temporaria* in 2008 and 2012, but significantly higher in all other years (Supplementary Data Table 1B).

The number of both live and dead *A. obstetricans* metamorphs encountered at Lac Arlet varied each year. 2010 saw the highest overall abundance of metamorphs, as well as demonstrating one of the highest proportions (0.44) of dead metamorphs (Figure 2b). A high number of *R. temporaria* metamorphs were encountered each year, but both *B. spinous* metamorphs and *A. obstetricans* OW larva steadily declined, with no *B. spinous* metamorphs being seen in 2013 or 2014 (Table 2). Five freshly dead *R. temporaria* metamorphs were collected in 2011 and 10 in 2012, all of which were examined histopathologically. Microscopic analysis revealed that one of five examined from 2011 and three of 10 examined from 2012 were demonstrably infected with *Bd*. The sections examined from two animals, one from 2011 and one from 2012, showed areas where a large proportion of epidermal cells were infected with epidermal thickening due to hyperkeratosis and parakeratosis. These findings are consistent with chytridiomycosis and, in the absence of any other abnormalities, indicate that this was the likely cause of death of these animals (Figure 2a).

The onset of spring, and therefore the length of the season of amphibian activity, varied over the years (Figure 1c; Table 1). We found a significant effect of onset of spring on the prevalence of *Bd* infection in both *B. spinosus* (Slope = -0.100; z = -4.715, d.f. = 149, *p* <0.001; adjusted R²=0.19), and *R. temporaria* (Slope = -0.039, z = -4.356, d.f. = 207, *p*<0.001; adjusted R²=0.08), with early spring onsets resulting in a higher prevalence of *Bd* infection (Figure 1d). We found no significant effect for *A. obstetricans* metamorphs (Slope = -0.029, z = -1.672, d.f. = 212, *p* = 0.09; adjusted R²=0.03). There was no significant association between the prevalence of infection in either *B. spinosus* or *R. temporaria* and the number of *A. obstetricans* mortalities seen (*p*= 0.95; 0.67 respectively), and the intensity of infection in *A. obstetricans* metamorphs (*p*= 0.68; 0.88 respectively), each year.

**Supplementary Data Table 1.** The differences in infection intensity (GE), by year, between **A)** *B. spinosus* and *R. temporaria* compared to *A. obstetricans* and **B)** between *B. spinosus* compared to *R. temporaria*. A lower (─) or higher (+) GE is indicated, along with the *p*-value for each result.

| A. |  | | *A. obstetricans* | | | | |
| --- | --- | --- | --- | --- | --- | --- | --- |
|  | 2008 | 2009 | 2010 | 2011 | 2012 | 2013 | 2014 |
| *B. spinosus* | ­**─** *p*<0.001 | **─** *p*<0.001 | **─** *p*<0.001 | **─** *p*=0.001 | **─** *p*<0.001 | NA | NA |
| *R. temporaria* | **─** *p*<0.001 | **─** *p*<0.001 | **─** *p*<0.001 | **─** *p*<0.001 | **─** *p*<0.001 | **─** *p*<0.001 | **─** *p*<0.001 |

| B. |  | | *R. temporaria* | | |
| --- | --- | --- | --- | --- | --- |
|  | 2008 | 2009 | 2010 | 2011 | 2012 |
| *B. spinosus* | ­**─** *p*<0.001 | +*p*<0.001 | +*p*=0.006 | +*p*<0.001 | **─** *p*=0.014 |

**Supplementary Data Figure Legends:**

**Supplementary Data Figure 1)** R-squared of linear regressions: of onset of spring against mean daily air temperature calculated over time periods of lengths varying from 21-81 days in 10 day increments, centred on days 30-170 of the year

**Supplementary Data Figure 2)** Current, and future mean daily temperatures (10-day moving average) for Lac Arlet across the whole year. Current temperatures are altitude-adjusted observations from nearby weather station. Future temperatures are means of 100 years of plausible weather generated by LARS-WG based on climate models GISS-E2 and HadGEM2, under RCP 8.5
